# The toxic effects and possible mechanisms of Brusatol on mouse oocytes

**DOI:** 10.1371/journal.pone.0177844

**Published:** 2017-05-18

**Authors:** Rujun Ma, Hongru Li, Yu Zhang, Ying Lin, Xuhua Qiu, Min Xie, Bing Yao

**Affiliations:** 1 Center for Reproductive Medicine, Jinling Hospital, Clinical School of Medical College, Nanjing University, Jiangsu, People's Republic of China; 2 College of Animal Sciences and Technology, Nanjing Agricultural University, Jiangsu, People's Republic of China; 3 College of Life Science, Nanjing Normal University, Jiangsu, People's Republic of China; China Agricultural University, CHINA

## Abstract

Brusatol is a natural quassinoid that shows a potential therapeutic use in cancer models by the inhibition of Nuclear factor erythroid 2-related factor 2 (Nrf2) and is capable of inducing a variety of biological effects. The effects of Brusatol on oocyte meiosis has not been addressed. In this study, we investigated the impact of Brusatol treatment on mouse oocyte maturation and its possible mechanism. Our data demonstrated that Brusatol treatment disrupted oocyte maturation and spindle/chromosome organization by modulating Nrf2-Cyclin B1 pathway, as the influence of Brusatol was compensated by the addition of Nrf2 activation plasmid, and the mRNA and protein levels of Cyclin B1 were severely reduced in oocytes following Nrf2 decline. In summary, our data support a model that Brusatol, through the inhibition of Nrf2, modulate Cyclin B1 levels, consequently disturbing proper spindle assembly and chromosome condensation in meiotic oocytes.

## Introduction

Oocyte quality is essential for female fertility. In mammals, oocytes development undergo germinal vesicle breakdown (GVBD), proper spindle assembly and polar body extrusion. During meiosis, microtubules organize into a barrel-shaped bipolar spindle, with chromatin condensation and all chromosomes aligned[1]. The oocytes proceed through the meiosis I (MI) division, then extruding the first polar body (Pb1), and arrested at metaphase II (MII), waiting for fertilization[2]. Any mistakes in this process should be defined as mutation, which is a major cause of pregnancy loss or severe birth defects[3].

Nrf2 is an important transcription factor that plays a critical role in the regulation of oxidative stress, aging-associated diseases and inflammation. Nrf2 mediates the induction of a battery of antioxidant defense enzymes, including NAD(P)H:quinone oxidoreductase-1 (NQO1), glutathione S-transferase (GST), γ-glutamate cysteine ligase catalytic subunit (GCLC) and so on, which are directly involved in protection against reactive oxygen species (ROS)[4]. Moreover, Nrf2 was regulated to modulate mitosis[5]. Several studies indicated that Nrf2 was also required for cell apoptosis and the expression of wee1, CDC2 and Cyclin B [6, 7]. Nrf2 deficiency caused a delay in hepatocyte proliferation, concomitant with dysregulation of the activation of Cyclin D1, E1, and A2[8]. The regulatory cascade with a hierarchy of p62–Keap1–Nrf2–NQO1 is required for p53 stabilization for mitotic catastrophe[9]. Although Nrf2 participate in multiple critical biological processes, to date, the precise roles of Nrf2 in mouse oocytes during meiosis have not been elucidated.

Brusatol is a member of quassinoids, which is acknowledged as a unique inhibitor of Nrf2-mediated signaling pathway, and acts by reducing the protein level of Nrf2 through inhibition of protein synthesis[10] and stimulation of its ubiquitination and proteolysis[11]. Although Brusatol shows a potential therapeutic use to combat chemoresistance in both in vitro and in vivo cancer models[11–13], the safety of Brusatol on fertility still needs to be studied.

The discovery of involvement of Nrf2 in meiosis of mouse oocyte, particularly in controlling meiotic progression suggesting a essential role in oocyte development. In this study, we proposed that Nrf2 would play important roles on mouse oocyte maturation. To confirm our hypothesis, Brusatol was used to investigate the effects of Nrf2 inhibition on mouse oocyte maturation.

## Materials and methods

All chemicals and culture media were purchased from Sigma (St. Louis, MO, USA) unless stated otherwise.

### Ethics statement of animals

6–8 weeks ICR female mice were used in this study. All mice were purchased from the Beijing Vital River Laboratory Animal Technology Co., Ltd (Beijing, China), and housed on a 12h/12h light/dark cycle at 22°C. All mice used in this study were physically normal and healthy. All experiments were approved by the Animal Care and Use Committee of Nanjing Jinling hospital and were performed in accordance with institutional guidelines.

### Antibodies

Rabbit polyclonal anti-Nrf2 (Cat#:ab137550) and Rabbit monoclonal anti-Cyclin B1 (Cat#:ab181593) were purchased from Abcam (Cambridge, MA, USA); Mouse monoclonal anti-α-tubulin-FITC antibody (Cat#:76074) was purchased from Sigma (St. Louis, MO, USA;); FITC-conjugated goat anti-rabbit IgG was purchased from Thermo Fisher Scientific (Rockford, IL, USA).

### Oocyte collection and culture

Approximately 46–48 h after injection of 5 IU Pregnant Mares Serum Gonadotropin (PMSG), fully-grown immature oocytes were harvested from ovaries of 6–8 week old female ICR mice which were sacrificed by cervical dislocation. Enclosed cumulus cells were removed by repeatedly pipetting, and then oocytes were cultured in M2 medium under mineral oil at 37°C in a 5% CO_2_ incubator. At appropriate time points, oocytes were selected for the following assays.

### Brusatol treatment

**Fig 1:**
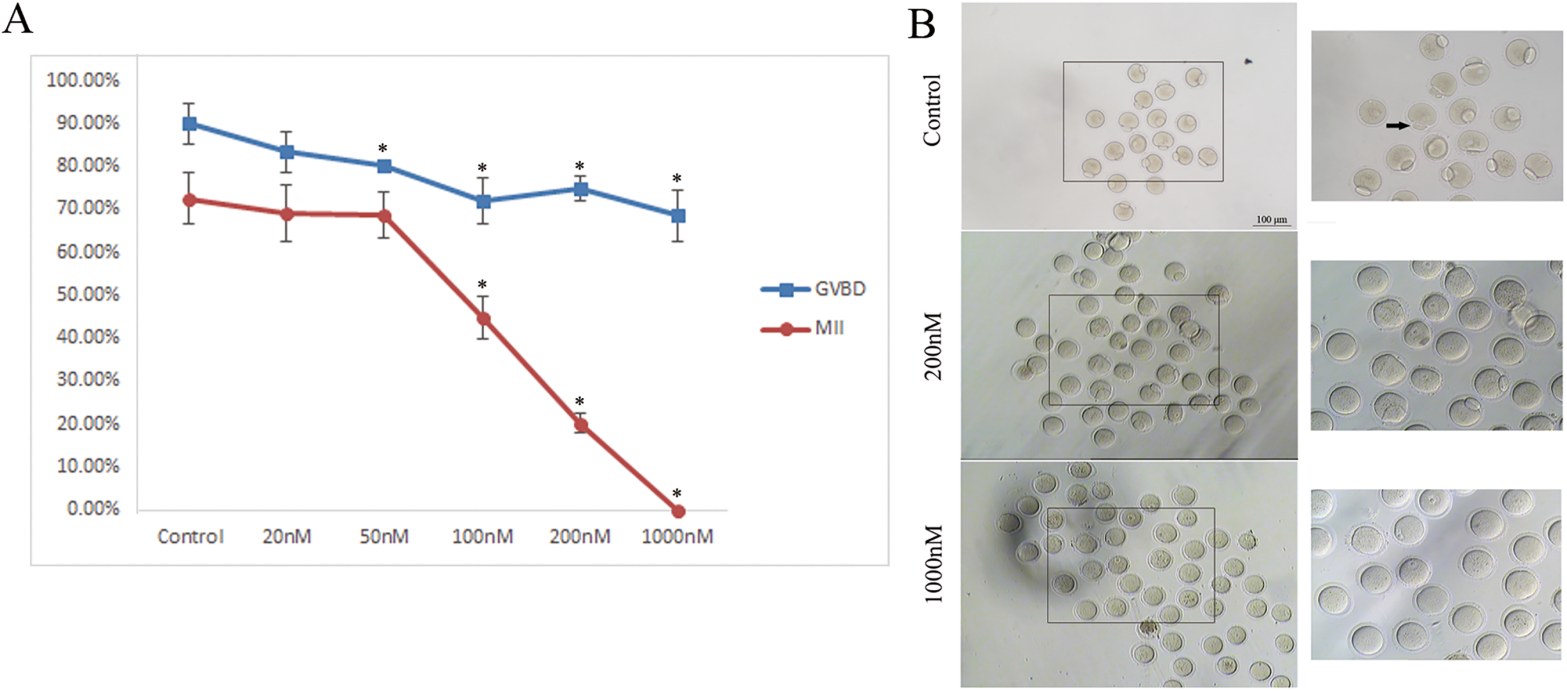
Effects of Brusatol on mouse oocyte maturation. A) Quantitative analysis of GVBD and Pb1 extrusion in different treatment groups (control, 20 nM, 50 nM, 100 nM, 200 nM and 1000 nM). B) Polar body extrusion failure after Brusatol treated. Images were acquired with a camera on a stereomicroscope. Arrows showed that the control oocytes extruded the polar body while the treated oocytes failed. Scale bar, 100 μm. *p < 0.05 vs controls.

Brusatol (Cat#:14907-98-3) was from Shanghai Tauto Biotech Co (Shanghai, CHN). A solution of Brusatol in DMSO (1 mM) was diluted in M16 medium to concentrations of 20, 50, 100, 200 or 1000 nM. The brusatol concentration of 200 nM had a significant effect on oocyte maturation were used for the experiments (Fig 1). Oocytes were then cultured in this medium for varying amounts of time and used for western blot and immunofluorescence microscopy. Controls were cultured with pure DMSO (1‰ vol/vol)at the same concentration under the same schedule. The Pb1 exclusion rates were examined under a stereomicroscope (IX73, Olmpus, Japan) equipped with the 10x or 20x objectives.

### Nrf2 activation

Nrf2 CRISPR Activation Plasmid (Cat#: sc-421869-ACT) was obtained from Santa Cruz Biotechnology (San Jose, CA, USA). Microinjection of Nrf2 CRISPR Activation Plasmid into the cytoplasm of fully-grown immature oocytes was used to overexpress Nrf2. Nrf2 CRISPR Activation Plasmid was diluted with water to give a stock concentration of 30 ng/μL, and then 2.5 picoliter solution was injected into oocytes with a Narishige microinjector. PBS was injected as control.

After injections, oocytes were arrested at germinal vesicle (GV) stage with 2.5 μM milrinone for 20 hours, and then were cultured in milrinone-free M16 medium for maturation.

### Quantitative real-time PCR

Total RNA was isolated from 50 oocytes using an RNA Isolation Kit (Life Technologies, Invitrogen TM, USA, Cat#: KIT0204), and cDNA was quantified by qRT-PCR using a Roche Light Cycler 96 Real-time PCR system (F. Hoffmann-La Roche Ltd, Basel, Switzerland). The fold change in gene expression was calculated using the ΔΔCt method with the house keeping gene, glyceraldehydes-3-phosphate dehydrogenase (GAPDH), as the internal control. Primer sequences are listed in S1 Table.

### Western blotting

A total of 100 oocytes at MI stage was collected, lysed in Laemmli sample buffer containing protease inhibitor, and boiled for 5 min before subjected to 10% SDS-PAGE for protein separation. A PVDF membrane was used to transfer the separated proteins for 1h at 275 mA at 4°C, then blocked in TBST (TBS containing 0.1% Tween 20) and 5% nonfat milk for 1 hour. Then the PVDF membrane was incubated overnight at 4^°^C with primary antibodies as follows: rabbit anti-Nrf2 antibody (1:1000), anti-Cyclin B1 (1:1000) and anti-actin antibody (1:2000). After 3 times washes in TBST, membranes were incubated with HRP-conjugated secondary antibodies for 1 hour at room temperature. Finally, the membranes were washed 3 times in TBST and then processed using an ECL Plus Western Blotting Detection System. This experiment was repeated at least 3 times using different samples.

### Measurement of intracellular ROS and mitochondrion

Intracellular reactive oxygen species (ROS) production was measured using CM-H2DCFDA (Life Technologies, Invitrogen TM, Cat#: C6827), mitochondrion was measured using MitoTracker Red CMXRos (Life Technologies, Invitrogen TM, Cat#: M7512). Oocytes were incubated with 5 mM CM-H2DCFDA or 200 nM MitoTracker Red CMXRos for 30 minutes at 37°C in a 5% CO_2_ incubator. After three washes, 5–10 oocytes were loaded on a slide with a microdrop of medium, and fluorescence images were recorded using a laser scanning confocal microscope (LSM 710; Carl Zeiss, Oberkochen, Germany).

### Immunofluorescence and confocal microscopy

For staining of Nrf2, Cyclin B1, tubulin and pErk1/2, oocytes were fixed with 4% paraformaldehyde for 30 minutes and then permeabilized with 0.5% Triton X-100 for 20 minutes. After 1 hour blocking in 1% BSA-supplemented PBS, samples were incubated overnight at 4^°^C with primary antibodies as follows: anti-Nrf2 antibody, FITC-conjugated anti-tubulin antibody and anti-Cyclin B1 antibody. After three washes in PBS, oocytes were labeled with goat-anti rabbit IgG or goat-anti mouse IgG at room temperature for 1 hour. Hoechst 33342 (blue) was used for chromosome staining. Oocyte samples were mounted on anti-fade medium (Vectashield, Burlingame, CA, USA), and then examined under a Laser Scanning Confocal Microscope (LSM 710, Zeiss, Germany) equipped with the 40x objectives.

### Statistical analysis

Data are presented as means ± standard deviations (SD), unless otherwise indicated. The means and SD of the data were calculated and statistically analyzed by Student’s t-test and analysis of variance (ANOVA) when appropriate (P < 0.05).

## Results

### Brusatol treatment disrupted meiotic progression in mouse oocytes

Oocyte maturation includes germinal vesicle breakdown (GVBD), microtubules organizing into the metaphase I (MI) spindle, chromosomes aligning at the the equator plate, execution of the MI division, extruding the first polar body (Pb1), and then proceeding to arrest at metaphase II (MII) waiting for fertilization. Our results showed that Brusatol affected GVBD and Pb1 extrusion rates in a dose-dependent manner in mouse oocytes (Fig 1A). Furthermore, 200 nM of brusatol dramatically decreased the GVDB rate after 3 hours culture, compared to controls (74.9 ± 2.9% vs. 90.1 ± 4.8% control, p < 0.05; Fig 1A), and the ratio of Pb1 extrusion after 14 hours in vitro-maturation (20.2 ± 2.2% vs. 72.6 ± 5.8% control, p < 0.05; Fig 1A and 1B). These data indicates that Brusatol infulences meiotic progression.

### Effects of Brusatol on spindle formation and chromosome organization in oocytes

**Fig 2:**
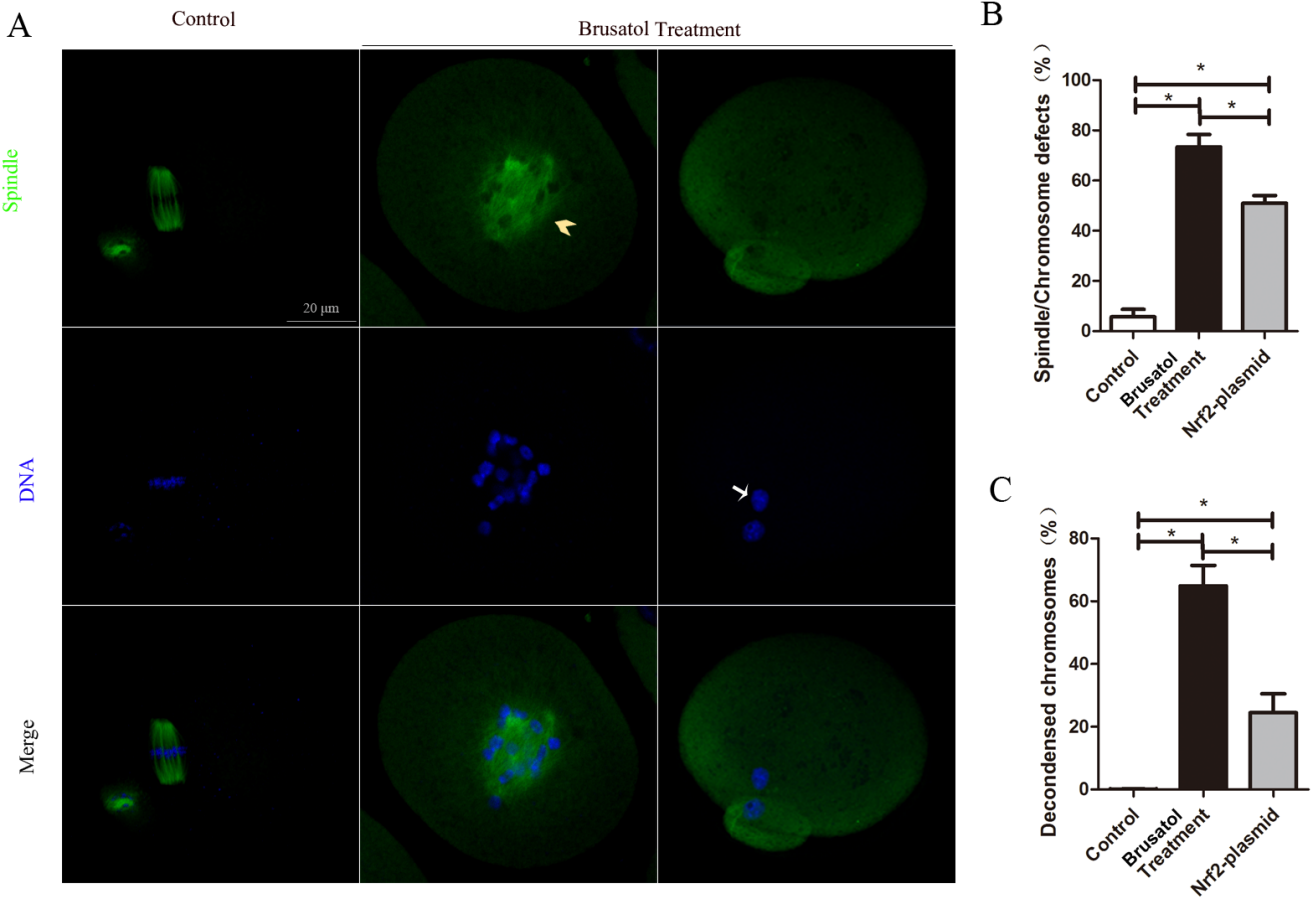
Brusatol treated results in abnormal spindles and chromosome organization defects. A) Oocytes at MII stage were immunostained with α -tubulin antibody to visualize spindle (green) and counterstained with Hoechst 33342 for chromosomes (blue). Representative confocal images showing the spindle morphology and chromosome alignment in control (a) and Brusatol treated (b-c) oocytes. Arrowheads indicate the abnormal spindle and arrows indicate the misaligned and decondensed chromosomes. B) Quantification of control, Brusatol treated and Brusatol+Nrf2-plasmid treated oocytes with spindle/chromosome defects. Data are expressed as mean percentage ± SD from three independent experiments in which at least 120 oocytes were analyzed. Scale bar, 20 μm. *p < 0.05.

To find out whether Brusatol treatment have effects on themeiotic apparatus in oocytes, oocytes were immunolabeled with anti-tubulin antibody to visualize the spindle and costained with Hoechst 33342 for chromosomes. Confocal microscopy revealed that Brusatol treated oocytes displayed a high frequency of spindle morphology defects and/or loose and misaligned chromosomes (73.5 ± 4.9% vs. 5.7 ± 3.1% control, p <0.05, Fig 2A and 2B). In particularly, after 14 h in vitro-maturation, Brusatol treated oocytes displayed high percentage of decondensed chromosomes (64.9 ± 6.5% vs. 0.1 ± 0.1% control, p <0.05, Fig 2C).

### Brusatol affects oocyte maturation via Nrf2 pathway

**Fig 3:**
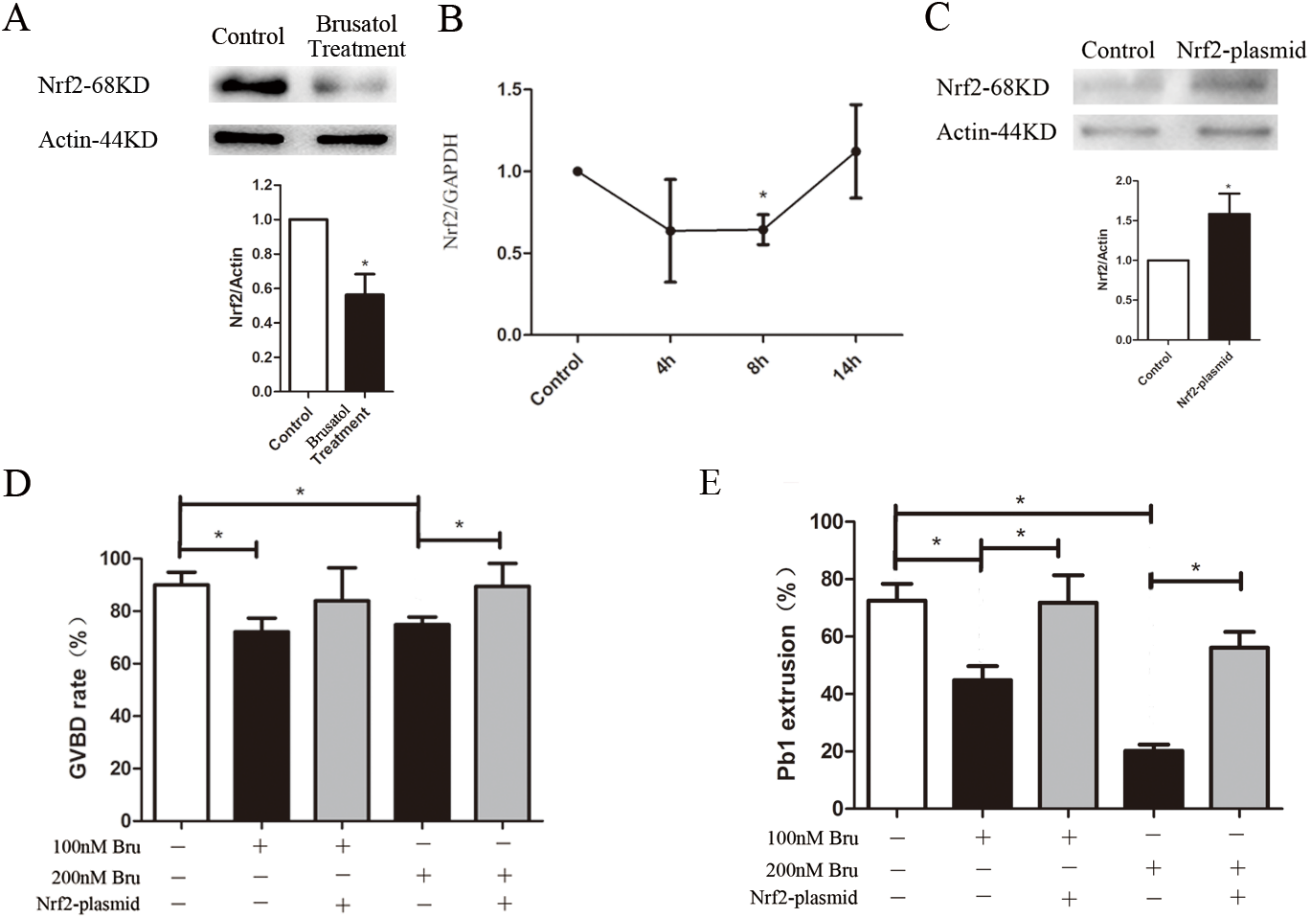
Brusatol affects oocyte maturation by Nrf2. A) Western blot showing decline of Nrf2 after Bruastol treatment with actin as a loading control. B) The relative mRNA level of Nrf2 was determined by qRT-PCR in control and Brusatol treated oocytes. mRNA level in control oocytes were set as 1. C) PBS (control group) or Nrf2 CRISPR Activation Plasmid (activation group) was microinjected into GV oocytes, which were arrested for 20 h with milrinone to allow synthesis of new Nrf2 protein. Then the oocytes were collected for Western blot. Results indicated that Nrf2 protein was efficiently overexpressed. D,E) Quantitative analysis of GVBD and Pb1 extrusion in different groups. Data are expressed as mean percentage ± SD from three independent experiments in which at least 80 oocytes were analyzed. *p < 0.05.

To confirm that Brusatol inhibits Nrf2, mouse oocytes were exposed to 200 nM Brusatol, and Nrf2 levels were determined by immunoblotting and realtime PCR. The treatment led to a significant reduction of Nrf2 protein from Brusatol treated oocytes, based on Western blot (Fig 3A). Although the mRNA level of Nrf2 was also declined in treated 8 h group, it had no difference with control at 14 h (Fig 3B). Then we performed overexpression experiments to test whether enhancing Nrf2 expression in Brusatol treated oocytes could rescue their meiotic phenotypes. Nrf2 was overexpressed by microinjection of Nrf2 CRISPR Activation Plasmid into full grown oocytes; PBS was injected as control. After injections, oocytes were arrested at the GV stage with milrinone for 20 h to allow synthesis of new Nrf2 protein. Then oocytes were washed and cultured with or without Brusatol until the appropriate time points to analyze meiotic progression. Immunoblotting confirmed that Nrf2 protein level was upregulated (Fig 3C), while the GVBD and Pb1 exclution rates were ameliorated in Brusatol treated oocytes (Fig 3D and 3E). We also assessed α-tubulin and chromosome in control oocytes and Brusatol treated oocytes, as well as Brusatol treated oocytes overexpressing Nrf2. Our data demonstrated that, compared to Brusatol treated oocytes, both spindle/chromosome defects (51.0 ± 5.2% vs. 73.5 ± 4.9% Brusatol treatment, p <0.05) and percentage of decondensed chromosomes (24.5 ± 6.0% vs. 64.9 ± 6.5% Brusatol treatment, p <0.05) were markedly lower in Nrf2 overexpression, as shown in Fig 2B and 2C. Taken together, these results suggested that Brusatol cause the meiotic defects by downregulation Nrf2.

### Brusatol selectively inhibits the Nrf2-mediated oxidative stress

**Fig 4:**
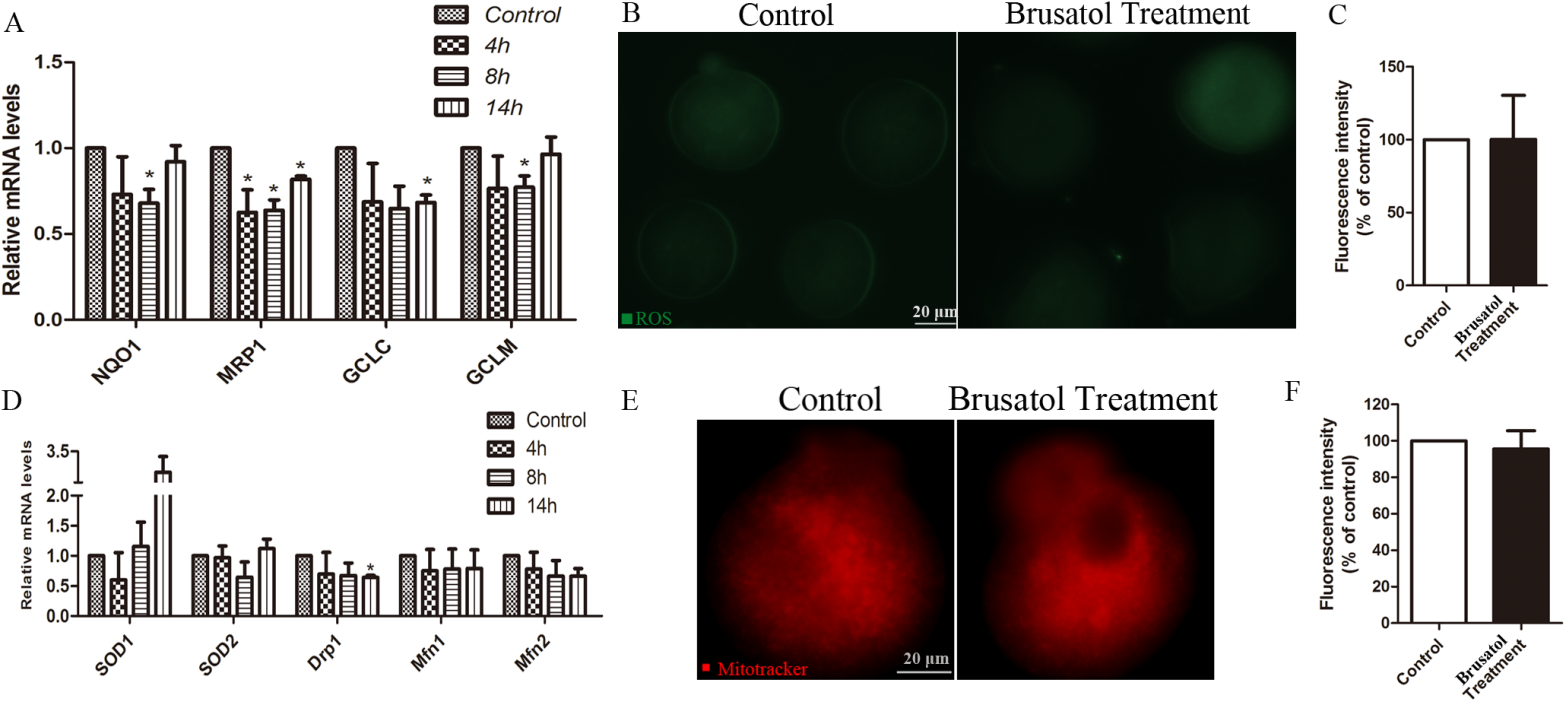
Effects of Brusatol treatment on oxidative stress and mitochondrion. A) Brusatol reduced the mRNA level of Nrf2 target genes. oocytes were treated with 200 nM of brusatol for 4h, 8h, 14h and subjected to qRT-PCR. B) Representative images of CM-H2DCFDA fluorescence in control, and Brusatol treated oocytes to show the ROS levels. C) Quantitative analysis of fluorescence intensity shown in B. D) Brusatol reduced the mRNA level of mitochondrial related genes. E) To mark the mitochondrion, representative images of mito-tracker fluorescence in control, and Brusatol treated oocytes were taken. F) Quantitative analysis of fluorescence intensity shown in E. Scale bar, 20 μm. *p < 0.05.

To test the possibility that Brusatol reduces Nrf2 through a posttranslational mechanism, mRNA expression of Nrf2, and Nrf2-target genes were measured by quantitative real-time PCR. Brusatol changed the mRNA level of Nrf2 (Fig 3B), decreased the level of Nrf2-target genes, including NQO1, MRP1, GCLC and GCLM (Fig 4A). To clear whether reduced Nrf2 and Nrf2-target genes expression can cause increased ROS production in Brusatol treated oocytes, we investigated ROS levels which showed no significant change (Fig 4B and 4C).

### Effects of Brusatol on mitochondrial function related genes in oocytes

Mitochondrion play an important role on spindle maintenance, inadequate redistribution of mitochondria may be one of the important factors contributing to spindle/chromosome disorganization[2]. Nrf2 is a prominent player in supporting the structural and functional integrity of the mitochondria[14]. We then asked whether the spindle defects in Brusatol treated oocytes were related with mitochondrion dysfunction. To address this question, we investigated the mRNA levels of mitochondrial-related genes, including SOD1, SOD2, DRP1, MFN1, MFN2. However, only DRP1 showed significant change (Fig 4D). Simultaneously, as shown in Fig 4E and 4F, the intensity of mitochondrial staining hasn’t changed significantly.

### Brusatol inhibited oocyte maturation by Nrf2 pathway though Cyclin B1

**Fig 5:**
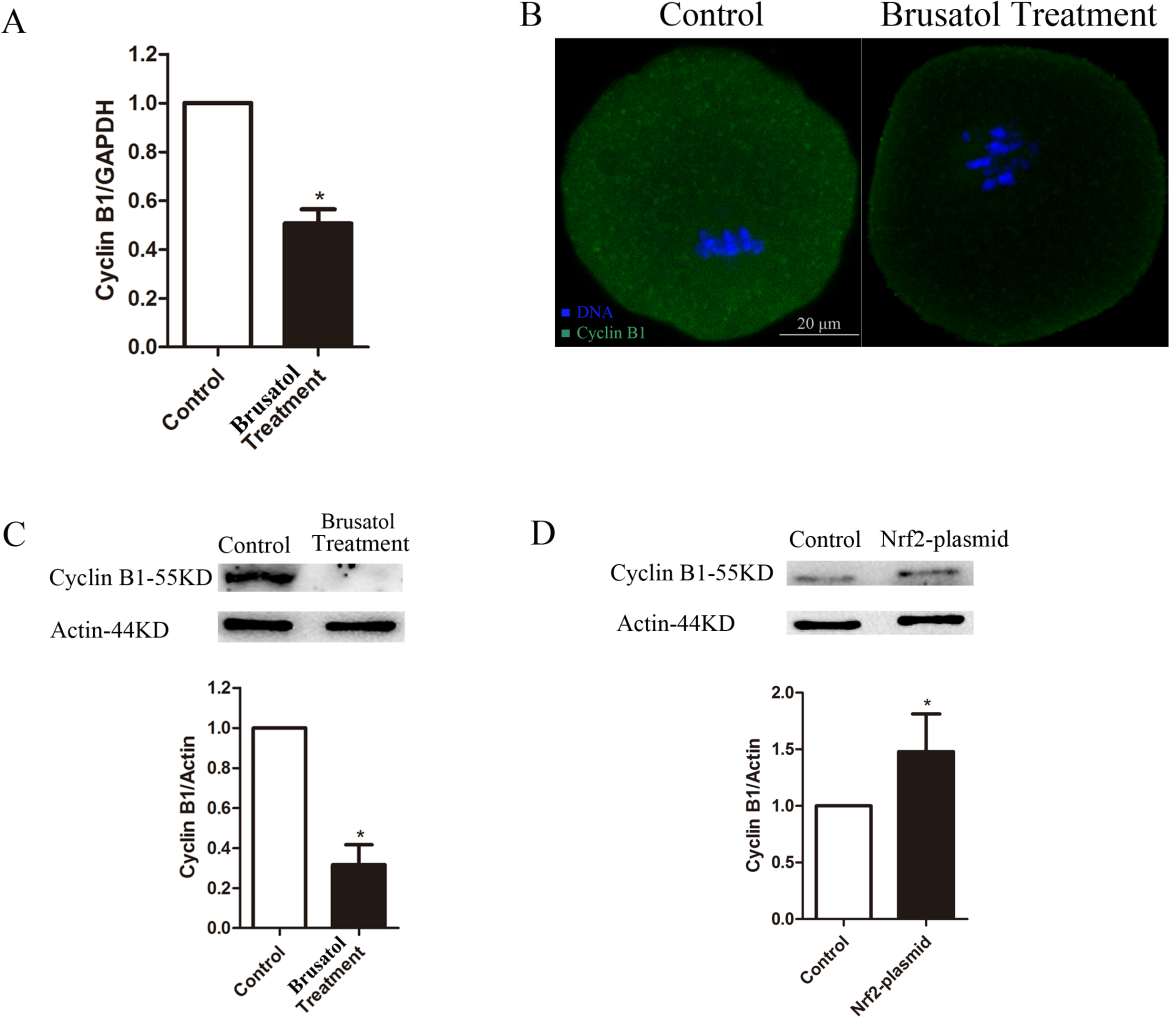
Brusatol reduces cyclin B1 expression in oocytes. A) The relative mRNA level of cyclin B1 was determined by qRT-PCR in control and Brusatol treated oocytes. mRNA level in control oocytes were set as 1. B) Oocytes at MI stage were immunostained with cyclin B1 antibody (green) and counterstained with Hoechst 33342 for chromosomes (blue). C) Western blot showing decline of cyclin B1 after Bruastol treatment with actin as a loading control. C) Western blot showing increase of cyclin B1 after Nrf2 overexpression with actin as a loading control. Error bars indicate ± sd Scale bar, 20 μm. *p < 0.05.

We then searched for a potential effector protein that would explain the requirement of Nrf2 for maintaining spindle morphology and chromosome condensation in oocytes. Of note, recent findings suggest that Nrf2 regulated cell cycle progression by adjusting a series of cyclin[6, 7, 15–17]. Cyclin B1 play an important role on G2/M cell cycle progression. We examined Cyclin B1 which associated with phenotypes and is likely to be affected by the Nrf2 downturn. As shown in Fig 5A–5C, compared with control oocytes, both mRNA and protein levels of Cyclin B1 were barely expressed in oocytes after Nrf2 inhibition. Furthermore, Cyclin B1 can be upregulated by Nrf2 overexpression (Fig 5D). These results suggest that Cyclin B1 is likely to be an effector protein for Nrf2 in oocytes.

## Discussion

Because brusatol can be used to enhance the efficacy of a wide variety of chemotherapeutic drugs to treat many types of cancers[11]. To assess the effect of brusatol on female reproductive capacity, this study presented here was designed to investigate the toxicity and preliminary mechanisms of Brusatol on mouse oocyte meiotic maturation. In the study, we showed that Brusatol treatment led to the pronounced loss of Nrf2 in mouse oocytes, which lead to oocyte maturation failure and spindle /chromosomal defects. Moreover, our data also demonstrated that the level of Cyclin B1 was regulated by Nrf2 in oocytes, which is critical for chromosome condensation and microtubule polymerization.

An inhibitory effect of Brusatol on the transcription factor Nrf2 is well established and has been demonstrated in diverse cell lines, such as A549, HT22, Hepa-1c1c7, βTC6 and RAW264.7[11, 18–21]. However, there is little information regarding the effects of Brusatol on mouse oocyte maturation. Similarly to previous studies, we found Brusatol exposure caused the inhibition of Nrf2 in mouse oocytes. As recent study showed that Brusatol exposure suppressed the cell proliferation in a time- and dose- dependent manner[22]. Our data showed a dose-response association of Brusatol exposure as for altering the maturation of mouse oocytes in vitro, both GVBD and Pb1 extrusion rates were affected. To investigate the mechanisms underlying the low oocyte maturation rate, we first tested the spindle/chromosome organization, which is necessary to produce a healthy oocyte. Our results showed that brusatol treatment disrupted spindle formation at Metaphase and then decondensed chromosomes after 14h treatment, which probably led to the failure of polar body extrusion. While the overexpression of Nrf2 can rescue abnormal phenotype in Brusatol treated oocytes, this indicated that Nrf2 contribute the effects on the oocyte maturation.

Nrf2, a redox-sensitive transcription factor, plays a critical role in the regulation of cellular defense against chemical and oxidative stress[23]. Therefore, we detected Nrf2-target genes, including NQO1, MRP1, GCLC and GCLM, which are related to antioxidant defense. After exposure of mouse oocyte to 200 nM brusatol for up to 14 h, these genes show time-dependent decreases in the mRNA levels, as described in mitotic cells[19]. Based on the literature [24–26], we postulated that ROS and/or mitochondria function are likely to be the targets of Nrf2 on spindle/chromosomes in oocytes. Although Nrf2-mediated signaling pathway was inhibited as described in mitotic cells, we unexpectedly discovered that Brusatol treatment had little effects on ROS production and mitochondria function in oocytes.

Instead, we discovered that loss of Nrf2 caused considerable reduction of Cyclin B1 levels in meiotic oocytes. Cyclin B1 synthesis and degradation regulated Maturation-promoting factor (MPF; cyclin-dependent kinase 1/cyclin B1) activity, which oscillates with oocytes entry and exit from meiosis I and meiosis II in mammalian oocytes[27, 28], and increased to drive germinal vesicle breakdown (GVBD), chromosome condensation, and microtubule polymerization in prophase I oocytes. Moreover, as previous reports, although Pb1 excluded, the oocytes failed to enter into the second meiotic M phase[27]. Of note, after cultured with 200 nM Brusatol for 14h, a high frequency of the chromosomes decondensed with microtubules depolymerization showed in mouse oocyte. It suggested that Nrf2 possibly effect the chromosome condensation. In support of this notion, the loss of Cyclin B1 were readily observed in metaphase oocytes treated by Brusatol. Furthermore, the increased expression of Cyclin B1 were observed in oocytes injected by Nrf2 plasmia, which further demonstrating that Nrf2 regulates Cyclin B1. Although the the relationship between Nrf2 and cyclin B deserve further study, these observations collectively suggest that Brusatol mediated Nrf2 ablation could disrupt the expression of Cyclin B1, and thereby contribute to GVBD failure, spindle morphology defects and decondensed chromosomes in oocytes.

In conclusion, oocyte quality is a critical element dictating the fertility of a female. This study reveals that the mouse oocytes maturation was disrupted after Brusatol treatment through spindle morphology and chromosome condensation, whichprovided the evidence for the toxic effects of Brusatol on reproductive systems.

## Supporting information

S1 Table Mouse primer sequences. (PDF)
