# Supplementary material for: The toxic effects and possible mechanisms of Brusatol on mouse oocytes
Source: PLoS One. 2017 May 18;12(5):e0177844. doi: 10.1371/journal.pone.0177844 (PMC5436816; doi:10.1371/journal.pone.0177844)
Supplement: S1 Table — (PDF) [file pone.0177844.s001.pdf]

S1 Table. Mouse primer sequences

Gene	Forward primer	Reverse primer
Nrf2	GTCTTCACTGCCCCTCATC	TCGGGAATGGAAAATAGCTCC
GAPDH	TCTTGCTCAGTGTCCTTGC	CTTTGTCAAGCTCATTTCCTGG
NQO1	TGAAGAAGAGAGGATGGGAGG	GATGACTCGGAAGGATACTGAAAG
MRP1	TGAACCATGAGTGTGCAGAAGGTG	TCACACCAAGCCAGCATCCTTGG
GCLC	ACCATCACTTCATTCCCCAG	TTCTTGTTAGAGTACCGAAGCG
GCLM	AATCAGCCCCGATTTAGTCAG	CGATCCTACAATGAACAGTTTTGC
SOD1	TGTGTCCATTGAAGATCGTGTG	TCCCAGCATTTCCAGTCTTTG
SOD2	TGCTCTAATCAGGACCCATTG	CATTCTCCCAGTTGATTACATTCC
Drp1	AGGTGGCCTTAACACTATTGACA	AGACGCTTAATCTGACGTTTGAC
Mfn1	CATTGCGTTTCGGTTTTTCCC	GAAGGAGCAGTAGGAGTTGAAG
Mfn2	TGAATGCTTCCCCTCTCAAG	TCCAGTTCTGTGTTCCTGTG
Cyclin B1	CTGACCCAAACCTCTGTAGTG	CCTGTATTAGCCAGTCAATGAGG
CDK1	ACAAAGGAACAATCAAACTGGC	AGCAACACTTCTGGAGATCG
